# An improved alignment-free model for dna sequence similarity metric

**DOI:** 10.1186/1471-2105-15-321

**Published:** 2014-09-28

**Authors:** Junpeng Bao, Ruiyu Yuan, Zhe Bao

**Affiliations:** Department of Computer Science and Technology Xi’an Jiaotong University, West Xianning Road, 710049 Xi’an, P.R. China

**Keywords:** DNA sequence similarity, Clustering, Alignment-free model, Classifications of nucleotide bases

## Abstract

**Background:**

DNA Clustering is an important technology to automatically find the inherent relationships on a large scale of DNA sequences. But the DNA clustering quality can still be improved greatly. The DNA sequences similarity metric is one of the key points of clustering. The alignment-free methodology is a very popular way to calculate DNA sequence similarity. It normally converts a sequence into a feature space based on words’ probability distribution rather than directly matches strings. Existing alignment-free models, e.g. k-tuple, merely employ word frequency information and ignore many types of useful information contained in the DNA sequence, such as classifications of nucleotide bases, position and the like. It is believed that the better data mining results can be achieved with compounded information. Therefore, we present a new alignment-free model that employs compounded information to improve the DNA clustering quality.

**Results:**

This paper proposes a Category-Position-Frequency (CPF) model, which utilizes the word frequency, position and classification information of nucleotide bases from DNA sequences. The CPF model converts a DNA sequence into three sequences according to the categories of nucleotide bases, and then yields a 12-dimension feature vector. The feature values are computed by an entropy based model that takes both local word frequency and position information into account. We conduct DNA clustering experiments on several datasets and compare with some mainstream alignment-free models for evaluation, including k-tuple, DMk, TSM, AMI and CV. The experiments show that CPF model is superior to other models in terms of the clustering results and optimal settings.

**Conclusions:**

The following conclusions can be drawn from the experiments. (1) The hybrid information model is better than the model based on word frequency only. (2) For DNA sequences no more than 5000 characters, the preferred size of sliding windows for CPF is two which provides a great advantage to promote system performance. (3) The CPF model is able to obtain an efficient stable performance and broad generalization.

## Background

With the rapid development of bioinformatics, the collected biologic data has become a giant monster and is still explosively growing. It is necessary to use data mining methods to analyze this tremendous data and find useful or interesting information from the data sets. Due to the extremely huge amount and complex structure of the data, sequence analysis of DNA and protein is a challenging issue in the bioinformatics field. There are many approaches proposed for the sequence analysis on DNA and protein. Among them, the Clustering approach is one of the most popular approaches because it requires less transcendental knowledge and need not mark the targets’ category before learning. After clustering, DNA sequence segments can be automatically divided into clusters to show their similarity in structure, which implies their functional similarity [1, 2]. Such a treatment has many benefits. For example, it is a powerful intelligent way to predict a genome’s function and learn the new world of bioinformatics. When an unknown genome is assigned to a known cluster, it can be convinced that the new genome may have the very similar function with others in the same cluster. But the DNA clustering quality can still be improved greatly. Since the DNA sequence similarity metric has a vital impact on the clustering result. We present a new DNA sequence similarity model to improve the DNA clustering quality.

The similarity of DNA sequence is a fundamental metric in bioinformatics, which is a basis for many applications including predicting unknown sequences’ functions or effects, constructing creatures(or species) phylogenetic tree, and analyzing homologous. Generally, there are two categories of DNA sequence similarity measuring approaches. One is alignment-based and the other is alignment-free. The alignment-based method directly compares two DNA sequences using string matching algorithms, such as BLAST [3], FASTA [4], UCLUST [5] and CD-HIT [6]. Obviously, it is a time-consuming process to match strings in a large scale database [7], and the violent changes of sequence lengths decline the performance of clustering.

The alignment-free method converts each piece of DNA sequence into a feature vector in a new space, in which the similarity can be quickly computed. Some alignment-free algorithms exploit probabilistic models to generate feature vectors, of which the Markov model [8–10] is extremely important and widely used in bioinformatic applications. However, there are some arguments on the Markov model. Deshpande and Karypis [11] reported that the SVM-based approaches are more effective than many traditional sequence classification algorithms, especially Markov model based techniques, in the DNA sequence comparison. Lu et al. [12] argued that the assumption of Markov model on the DNA sequences impairs its capability.

Qi et al. [13] proposed a comparison method based on the probability of appearance of K-Strings. In order to suppress single-sequence noises, Reinert et al. [14] proposed  and  for sequence comparison based on k-tuple content. They are two variants of the *D*_2_ word count statistic. The *D*_2_ based alignment-free models measure the difference between two word probability distributions for sequence comparison. It is a widely used statistics method for sequence comparison based on the joint k-tuple content in the two sequences. Bauer et al. [15] confirmed the existence of a species specific Average Mutual Information (AMI) profile and took these profiles to measure the evolutionary relationships between genomic sequences.

The k-tuple algorithm [16, 17] is a very popular alignment-free method. It segments a DNA sequence via a sliding window of length *k*. A segment of DNA sequence in the window is a tuple, usually called a word of length *k*. The k-tuple algorithm counts the frequency of each tuple, i.e. word, to build a feature vector based on the frequency value. Since a DNA sequence is converted into a fixed length vector, it can be quickly measured by some distance metric.

It has been proved in many researches that the simple k-tuple method cannot completely describe all information contained in a DNA sequence, since it only contains the word frequency information. Therefore, many modified k-tuple algorithms are proposed to contain more information in models. Liu et al. [18] appended the mean and variance value of each word’s position distribution into the feature vector. As a result, the size of the final feature vector becomes three times of 4^*k*^. This method increases the information contained in a feature vector at the expense of computing overhead, in terms of larger processing latency and memory. Wei et al. [19, 20] presented a Distance Measure based k-tuples (DMk) method for DNA sequence clustering. According to the position distribution of each word, the DMk method calculates its entropy value to construct a feature vector. Dai et al. [21] utilized both the word frequency and overlapping structure of words to improve the efficiency of sequence comparison. Li and Wang [22] counted the information of codon positions, and calculated the relative entropy over 12-dimension feature vectors to discriminate protein coding and non-coding sequences in the yeast genome. Wang and Zheng [23] presented the Weighted Sequence Entropy (WSE) based comparison on word frequencies to modify the classical relative entropy. Zhao et al. [24] transformed the DNA sequence into the 60-dimension distribution vectors. Lu et al. [12] summarized the word frequency information over a serial of sliding windows with their size varying from 1 to *k*. Consequently, they have to observe all k-mer strings’ probability, and the amount of sub-strings is up to 4^*k*^.

The key issue of the alignment-free method is that various DNA characteristics and features should be integrally considered and carefully composed so as to contain sufficient original DNA information in the converted feature space. Shi and Huang [25] proposed a Three Sequence Method (TSM) to build a twelve-component feature vector. Yu [26] converted a DNA sequence into three 2-dimension cumulative ratio curves instead of symbolic sequences. Li and Wang [27] presented a 16-dimension binary vector based on the group of nucleotide bases.

A segment of DNA data can be considered as a text written by using a four-letters alphabet. So some researchers apply text clustering methods to DNA data, such as [2, 28–30]. It is confirmed that key words are flocking and not randomly distributed in DNA sequences [2, 28].

In this paper, we present an improved alignment-free model, named as CPF model, which combines advantages of other algorithms, such as k-tuple [16, 17], DMk [20] and TSM [25]. The CPF model converts a DNA sequence into three new sequences according to the classification of nucleotide bases, takes both frequency and position distribution information into account, and measures the similarity in a 12-dimension space. Thus, the CPF model contains more information than traditional alignment-free algorithms and achieves better clustering quality. The experimental results demonstrate the effectiveness of the CPF model.

## Methods

### Motivation

Our direct motivation is to improve the DNA clustering quality rather than detect homologous sequences. It is well known that the basic k-tuple method only containing word frequency information is not sufficient to fully describe a DNA sequence. Unsufficient information in a feature vector is the most important reason that causes poor clustering results. For instance, Dong and Pei [31] argued that the position inside sequence is important information for the sequence data clustering or classification. Thus, many modified algorithms adopt the position information of nucleotide bases. Among them, the DMk method [20] considers the occurrence, location and order relation of k-tuple in a DNA sequence. It produces a feature vector based on the Shannon entropy that reflects the degree of importance of positions in a sequence, instead of simply adding new statistical information. The vector size of DMk is equal to that of k-tuple under the same sliding window size. However, the DMk method ignores the classifications of nucleotide bases, which are very useful information to discriminate DNA pieces.

Many researches show that the classification over nucleotide bases improves the efficiency when comparing DNA sequences. TSM [25] converts a DNA sequence into three symbolic sequences utilizing the classifications of nucleotide bases and their chemical properties. TSM benefits from such a treatment. But the improvement of TSM is limited because it only involves the classification and word frequency information, while the position information is not included.

In this paper, we aim at using the word frequency, position and nucleotide bases classification in calculating the DNA sequence similarity. We integrate them together to enrich the feature vector but without big dimensions. This idea inspired us to design a new model, named *Category-Position-Frequency* model (CPF).

### The feature vector space

According to the chemical properties of nucleotide bases, they can be divided into three categories,

1. purine group *R*={*A*,*G*} and pyrimidine group *Y*={*C*,*T*};
2. amino group *M*={*A*,*C*} and keto group *K*={*G*,*T*};
3. weak hydrogen bond group *W*={*A*,*T*} and strong hydrogen bond group *S*={*C*,*G*}.

In terms of a specific category, each nucleotide base is mapped to a group. Hence a DNA sequence is mapped to a new alphabet space that is shrunk from 4^*k*^ to 2^*k*^. In this paper, we set the size of sliding window as 2, which is the preferred value verified by our experiments. Thus, the length of a word is 2 so that a mapped sequence contains 2^2^=4 words. Consequently, three categories produce twelve different words, which form our feature vector space. Namely, each dimension represents a word as follows.
1

**Figure 1:**
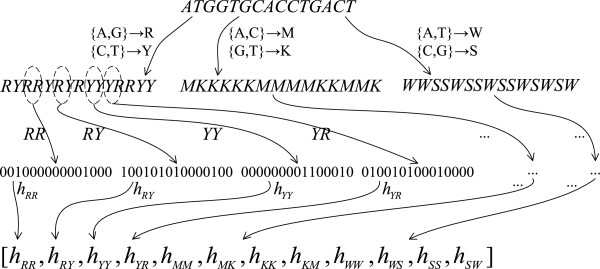
**The construction process of a DNA sequence feature vector.**

The Figure 1 illustrates the construction process of a DNA sequence feature vector.

### The feature value

Instead of the frequency of the word in the mapped sequence, we set the value of each dimension equal to the Shannon Entropy of words. The Shannon Entropy can expose the importance of the position distribution in the word [22]. A non-negative sequence *X*={*x*_1_,*x*_2_,…,*x*_*n*_} produces a sequence of its partial sum *S*, i.e.
2

Let *Z* denote the sum of the whole sequence *S*, i.e.
3

Obviously, the smaller the position of *x*_*r*_, the more it contributes to *Z*. Because the element *x*_*r*_ at the position *r*(*r*=1,2,…,*n*) is summed (*n*−*r*+1) times, i.e., the preceding element has a larger weight than *x*_*r*_. It implies that the partial sum based model prefers the element at a small position. Then the discrete probability at the position *r*, denoted by *p*_*r*_, is defined as follows.
4

The entropy of the sequence *X* can be calculated as
5

This entropy is able to reflect the importance of position in a sequence. Indeed, the front position tends to have a larger entropy. The biggest value of entropy is *log*_2_*n*, where *n* is the length of the sequence.

Li and Wang [22] make the original sequence from the word frequency at some positions. The frequency, termed Global Frequency, is counted over the whole sequence. Wei et al. [20] propose another frequency, called the Local Frequency, by counting the distance between two positions where a word occur twice.
6

where  is the local frequency, *r* is the order of occurrence of a word *w*,  denotes the position of the *r*th occurrence of the *w*, and  is defined as 0. LF emphasizes the position of occurrence and the local density of a word. A single LF cannot contain the global information of the word. But a sequence of LFs can show the word’s global distribution more precisely and clearly than the GF. In this paper, we exploit the LF to make the original nonnegative sequence.

The Table 1 illustrates examples to show the differences between entropy value and LF based entropy value. The length of the example sequences is eight so that the range of entropy is [0,3], where the sequence “10000000” gets the biggest value, and “00000001” gets the smallest. When the sequences are regularly shifting from “10000001” to “11000000”, the LF based entropy values keep a consistent trend, but the entropy values are fluctuant. It is believed that the LF based entropy reflects more subtle structural information than basic entropy.

**Table 1:** **The entropy values and LF based entropy values of example sequences**

Original	Partial sum	Entropy	Local frequency	LF based partial sum	LF based entropy
sequence***X***	sequence***S***,	value h	sequence***LF***,	sequence***S***_***LF***_,	value***h***_***LF***_,
	***S←X***	***h←S***	***LF←X***	***S***_***LF***_ ***←LF***	***h***_***LF***_ ***←S***_***LF***_
10000000	11111111	3	10000000	11111111	3
00000001	00000001	0			0
10000001	11111112	2.9477			2.9985
10000010	11111122	2.9219			2.9966
10000100	11111222	2.6186			2.9942
10001000	11112222	2.5546			2.9911
10010000	11122222	2.5388			2.9868
10100000	11222222	2.6154			2.9808
11000000	12222222	2.9736	11000000	12222222	2.9736

Finally, a DNA sequence’s feature vector is made from the LF based entropy according to the Eq. 1. The similarity between sequences is measured by the Euclidean Distance of feature vectors, i.e.
7

where *d*_1_ and *d*_2_ are two DNA sequences, *H*(*d*_1_) and *H*(*d*_2_) denote their feature vectors respectively.

### The pseudo code

The following is the pseudo code of the CPF model.


### The time and space complexity

The entire clustering process has two stages, first it makes the feature vectors from the raw DNA sequences, and then it runs the clustering algorithm. At the first stage, the CPF time complexity of making a feature vector is , where *n* is the length of a DNA sequence, *k* is the length of a sliding window,  is the average count of a word in a sequence. It is assumed that the occurrence probability of each word is equal to each other. Hence, the average count of a word is . Therefore, the CPF time complexity of making a feature vector is *O*(15*n*), i.e.
8

At the second stage, the CPF model runs a clustering algorithm in a 12-dimension feature space because the preferred size of sliding window is two. The time and space complexity depend on the specific clustering algorithm. It is well known that the time complexity of the standard k-means is *O*(*Icdn*), where *I* is the number of iterations, *c* is the number of clusters and *d* is the dimensions of the feature vector. As a result, when the CPF model runs the standard k-means clustering algorithm, the total time complexity of the two stages is *O*(15*n*+12*Icn*).

The CPF space complexity of making a feature vector is . According to the above assumption, it equals *O*(7*n*). When the CPF is implemented by a serial program without any parallel processing, the first stage time complexity on the whole dataset is *O*(15*n*×|*D*|) where |*D*| denotes the number of DNA sequences in the dataset *D*. And the space complexity is still *O*(7*n*).

## Results

### Experiment settings

We use the k-means algorithm, which is implemented by the scipy module in Python, to test our CPF model and compare it with other five alignment-free models, i.e. k-tuple, DMk, TSM, AMI [15] and CV [13]. We also compare the CPF based k-means with UCLUST and CD-HIT, which are two alignment based DNA clustering models.

Seven datasets DS2, DS3, DS4, HOG20, HOG50, HOG80 and HOG100 are collected from PBIL [32]. The DS2 dataset is the HOVERGEN from PBIL, which is a database of homologous vertebrate genes. The DS4 is randomly selected from HOMOLENS, which is a database of homologous genes from Ensembl organisms and Ensembl families. The rest are randomly selected from HOGENOM, which contains homologous gene families from microbial organisms.

Each DS* dataset, which is also used by Wei et al. [20], contains six families. Each HOG* dataset contains much more families that are varying from 20 to 100. The Table 2 lists the details of these datasets.

**Table 2:** **The details of the seven datasets**

Dataset	Number of	Total number of DNA	Average length of a DNA	Size of
	families	sequences in the dataset	sequence in the family	dataset (KB)
DS2	6	285	1307	396
DS3	6	310	1536	501
DS4	6	251	1075	291
HOG20	20	1542	1492	2488
HOG50	50	3327	1466	5285
HOG80	80	7305	1413	11207
HOG100	100	9648	1484	15501

The goal of the test is to divide the DNA sequences that belong to the same family into the same cluster as well as possible. Though the data origin is clear, no clustering algorithm can precisely and correctly re-arrange all data into the correct clusters. As well known, the initial cluster centers, which are randomly selected in the k-means algorithm, have a great effect on the results. In fact, the k-means clustering results are varying every time because of the random initial cluster centers. In order to eliminate the occasional disturbance, we repeat each experiment 10 times to count its average performance. At last, all models are evaluated in terms of average purity and F-measure, which are defined as follows.

Let *M* denotes the number of families in the dataset,  denotes the number of clusters in the whole clustering result, |*D*| denotes the number of the total DNA sequences in the dataset, *N*_*i*_ denotes the number of sequences in the family *i*,  denotes the number of sequences in the cluster *j*, *N*_*ij*_ denotes the number of sequences that belong to both family *i* and cluster *j*. *lbl*(*j*) denotes the familiy label of the cluster *j*, i.e. most members in the cluster *j* belong to the familiy *lbl*(*j*).

The purity of the cluster *j* is defined as:
9

The purity of the whole clustering result is:
10

Usually, the members in a cluster may be from several families. But the cluster is labelled by the dominated members. The familiy label of the cluster *j* is:
11

Since a family is often divided into serval clusters. We count the precision and recall of a family over the clusters that have the same family label. The precision of the familiy *i* is:
12

The recall of the familiy *i* is:
13

where *m*_*i*_ dontes the number of clusters whose familiy labels are equal to *i*.

The F-measure of the familiy *i* is:
14

The F-measure of the whole clustering result is:
15

### Clustering results

Since the k-means clustering results depend on the number of initial cluster centers, which is denoted by *c* value in the following. We vary *c* value to observe the performance variation tendency of models.

It is noted that the sliding windows size of CPF and TSM is 2, the rest is 3. When a feature vector is created from a DNA sequence, the *k* value, i.e. the size of the sliding window, has a significant effect on it. It is well known that a DNA sequence is composed of four letters alphabet {A, C, G, T}. Hence the size of the feature vector is 4^*k*^. Nevertheless, it is not always true that the bigger *k* value the better it is. Wei et al. [20] suggested that *k* value should be set 3 because the length of a DNA codon is 3. They believe that this value is helpful to conserve the inherited information in a DNA sequence. For using the same token, it is also set 3 in DMk, k-tuple, AMI and CV. Aita et al. [33] attempted to optimize the *k* value according to a mathematical model of mutational events. Although the CPF model transforms a DNA sequence into three new sequences, the newly generated sequences are composed of only two letters. Consequently, we assign the sliding window size to 2 in CPF and TSM. The experimental results show that it is suitable for CPF model.

**Figure 2:**
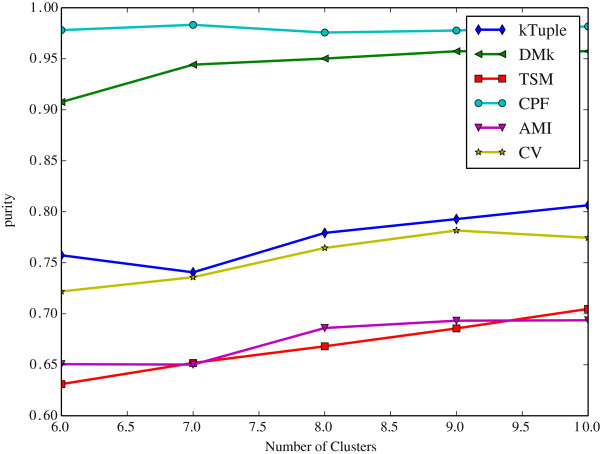
**The clustering results measured in purity against the number of initial clusters on the dataset DS4.**

**Figure 3:**
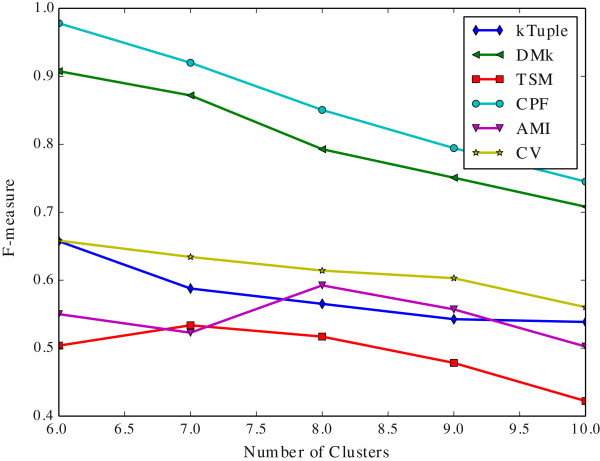
**The clustering results measured in F-measure against the number of initial clusters on the dataset DS4.**

**Figure 4:**
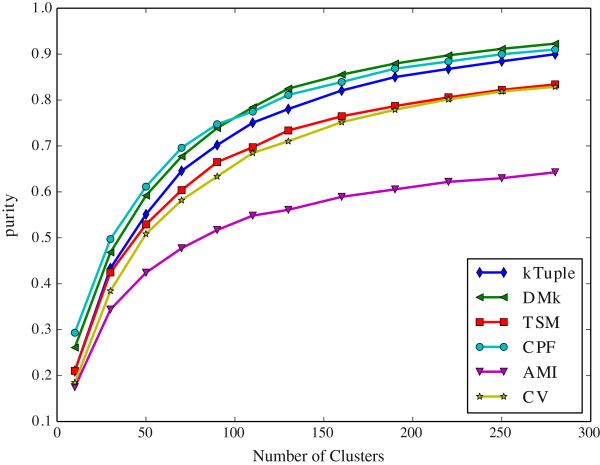
**The clustering results measured in purity against the number of initial clusters on the dataset HOG50.**

**Figure 5:**
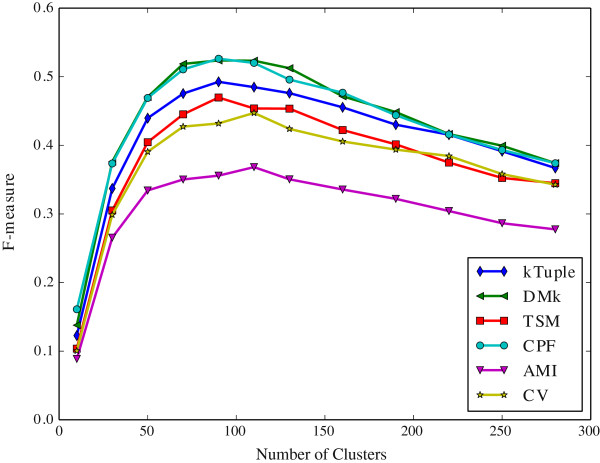
**The clustering results measured in F-measure against the number of initial clusters on the dataset HOG50.**

The Figure 2 illustrates the clustering results measured in purity against the number of initial clusters on the dataset DS4. The Figure 3 illustrates the same clustering results measured in F-measure on the same dataset. The Figure 4 illustrates the clustering results measured in purity against the number of initial clusters on the dataset HOG50. The Figure 5 illustrates the F-measure results on the dataset HOG50. The variation tendencies of cluster results on the dataset DS2 and DS3 are similar to the DS4. The tendencies on the HOG* datasets are similar to that of HOG50. On the HOG50 dataset, we vary *c* value from 10 to 280.

All models achieve the peak F-measure value in the range [50-150] whereas the purity value tends to be flat when the *c* value is greater than 50. Obviously, the bigger *c* value, the more cluster numbers, and the smaller a cluster is. A smaller cluster certainly has a higher purity(i.e. precision) but a lower recall. A cluster that contains only one sequence will get the highest purity and the lowest recall. But it is meaningless to our test because our goal is to put the similar DNA sequences together and ensure that they are from the same family. So the F-measure assesses clustering results more comprehensive than purity.

In General, the CPF is the best in most cases, and DMk is slightly worse than CPF, but it is much better than the rest models. TSM, k-tuple, AMI and CV are about the same level in most cases. For the F-measure, the CPF achieves the best result when *c* value is equal to or slightly greater than the number of families in the dataset,

The Table 3 lists the best clustering results in F-measure of CPF and two alignment based models, i.e. UCLUST and CD-HIT, on different datasets. The two alignment based models are far worse than CPF. However, UCLUST and CD-HIT are designed for finding the most similar DNA sequences, namely, every sequence in the cluster must have similarity above a given identity threshold (*T*). So these two alignment based models always get plenty of small clusters, which leads to a high precision (i.e. purity) score but a very poor recall score. As a result, their F-measure scores are worse. For example, on the HOG50 dataset, the UCLUST outputs 484 clusters, and the CD-HIT outputs 625 clusters, though all DNA sequences are from 50 families.

**Table 3:** **The best clustering results in F-measure of CPF and alignment based models on different datasets**

	UCLUST		CD-HIT		CPF	
Dataset	F-measure	Number of	F-measure	Number of	F-measure	Number of
		cluster		cluster		cluster
DS2	0.0623	197	0.2429	44	0.9755	6
DS3	0.0414	285	0.0620	189	0.9809	6
DS4	0.0633	183	0.1241	127	0.9761	6
HOG20	0.2590	197	0.2287	246	0.7791	20
HOG50	0.2197	484	0.1652	625	0.5576	50
HOG80	0.1871	897	0.1648	1033	0.5024	80
HOG100	0.1804	1185	0.1533	1433	0.4780	100
Settings	0.75≤*T*≤1		0.8≤*T*≤1		*k*=2	

Based on above evaluation, CPF model performs stably and consistently with various datasets. It is very easy to set optimal configurations for CPF, which implies that the CPF model has a good capability of generalization and applicability.

### The preferred sliding window size

The sliding window size, i.e. *k* value, determines the dimension of feature vector. The longer sliding windows size, the bigger feature vector dimension is. We vary the size of sliding window from 2 to 6 to observe its influence on the alignment-free models, while the number of k-means initial clusters is fixed on the number of families in the dataset.

**Figure 6:**
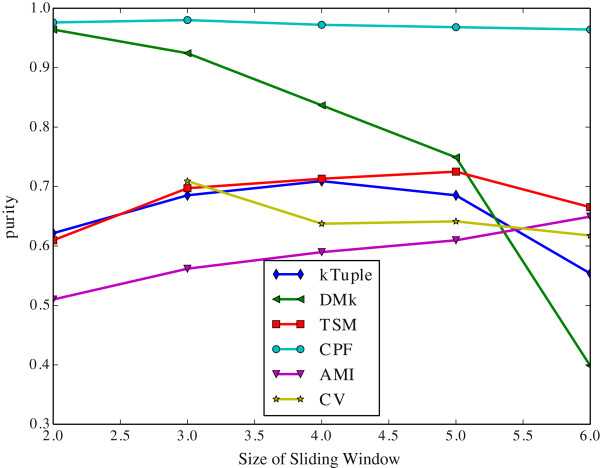
**The clustering results measured in purity against sliding window size on the dataset DS4.**

**Figure 7:**
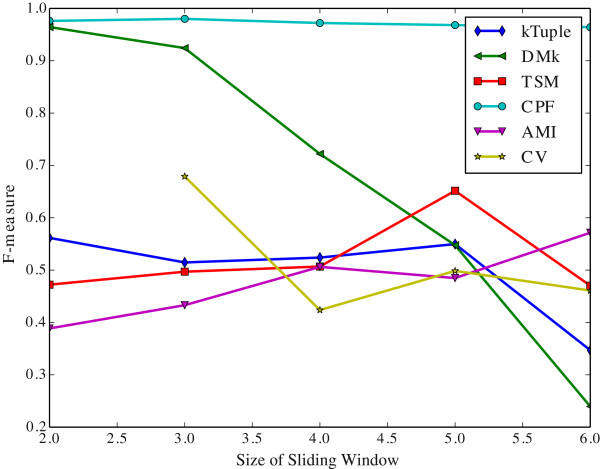
**The clustering results measured in F-measure against sliding window size on the dataset DS4.**

**Figure 8:**
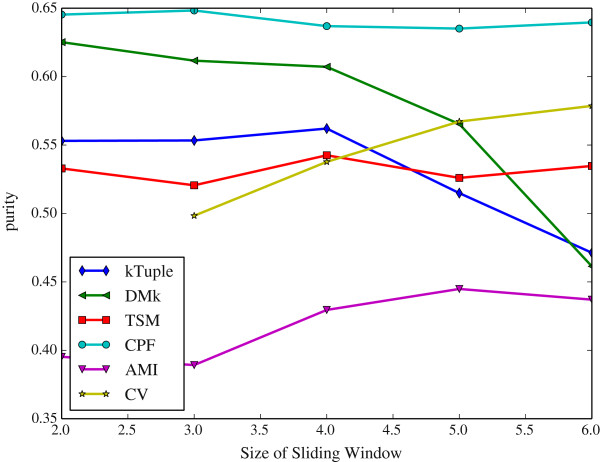
**The clustering results measured in purity against sliding window size on the dataset HOG50.**

**Figure 9:**
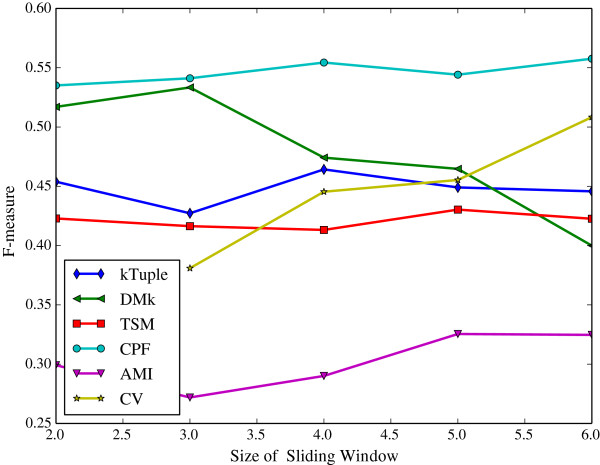
**The clustering results measured in F-measure against sliding window size on the dataset HOG50.**

The Figure 6 illustrates the clustering results measured in purity against the size of sliding window on the dataset DS4. The Figure 7 illustrates the same clustering results measured in F-measure on the same dataset. The Figure 8 illustrates the clustering results measured in purity against the size of sliding window on the dataset HOG50. The Figure 9 illustrates the F-measure results on the dataset HOG50. The variation tendencies of cluster results on the DS* datasets are similar to that of DS4. The tendencies on the HOG* datasets are similar to that of HOG50. The Tables 4 and 5 list the details on all datasets.

**Table 4:** **The clustering results of six alignment-free models against sliding window size on the dataset DS2, DS3 and DS4**

Dataset	Assessment method	Model	Size of sliding window				
			2	3	4	5	6
DS2	purity	kTuple	0.8842	0.9123	0.8947	0.7789	0.7404
		DMk	0.9474	0.9404	0.9474	0.8667	0.5123
		AMI	0.5895	0.6035	0.6140	0.5614	0.5965
		CV	N/A	0.7158	0.7684	0.8421	0.8421
		TSM	0.8667	0.8702	0.8772	0.8807	0.9018
		CPF	0.9754	0.9439	0.9368	0.9404	0.9123
DS2	F-measure	kTuple	0.8921	0.9184	0.9012	0.6631	0.6381
		DMk	0.9487	0.9419	0.9490	0.7477	0.3854
		AMI	0.4871	0.4924	0.5009	0.4104	0.4370
		CV	N/A	0.6126	0.7708	0.8457	0.8419
		TSM	0.8749	0.8789	0.8845	0.8878	0.9087
		CPF	0.9755	0.9451	0.9379	0.9416	0.9158
DS3	purity	kTuple	0.5935	0.6290	0.6290	0.5452	0.4968
		DMk	0.8968	0.8806	0.8774	0.7484	0.5774
		AMI	0.5387	0.5484	0.5452	0.5516	0.5581
		CV	N/A	0.4484	0.5419	0.5935	0.4452
		TSM	0.6290	0.6032	0.5774	0.5774	0.6452
		CPF	0.9806	0.9419	0.9194	0.9387	0.9226
DS3	F-measure	kTuple	0.4755	0.5158	0.5185	0.3862	0.3952
		DMk	0.8972	0.8836	0.8811	0.6336	0.3083
		AMI	0.4241	0.4699	0.4871	0.4284	0.4936
		CV	N/A	0.3473	0.4272	0.4816	0.2307
		TSM	0.5046	0.4871	0.4208	0.4224	0.5364
		CPF	0.9809	0.9446	0.9222	0.9420	0.9262
DS4	purity	kTuple	0.6215	0.6853	0.7092	0.6853	0.5538
		DMk	0.9641	0.9243	0.8367	0.7490	0.3984
		AMI	0.5100	0.5618	0.5896	0.6096	0.6494
		CV	N/A	0.7092	0.6375	0.6414	0.6175
		TSM	0.6096	0.6972	0.7131	0.7251	0.6653
		CPF	0.9761	0.9801	0.9721	0.9681	0.9641
DS4	F-measure	kTuple	0.5616	0.5146	0.5240	0.5497	0.3469
		DMk	0.9644	0.9242	0.7220	0.5476	0.2395
		AMI	0.3886	0.4330	0.5062	0.4848	0.5714
		CV	N/A	0.6790	0.4237	0.4986	0.4610
		TSM	0.4722	0.4970	0.5068	0.6516	0.4703
		CPF	0.9761	0.9801	0.9721	0.9681	0.9641

**Table 5:** **The clustering results of six alignment-free models against sliding window size on the dataset HOG20, HOG50, HOG80 and HOG100**

Dataset	Assessment method	Model	Size of sliding window				
			2	3	4	5	6
HOG20	purity	kTuple	0.6401	0.6505	0.6388	0.5986	0.5253
		DMk	0.7808	0.7646	0.7639	0.6900	0.5674
		AMI	0.5649	0.5765	0.5817	0.5798	0.5798
		CV	N/A	0.6064	0.5934	0.6206	0.6595
		TSM	0.6174	0.6206	0.6329	0.6297	0.6128
		CPF	0.8119	0.7970	0.8113	0.7879	0.7737
HOG20	F-measure	kTuple	0.5810	0.5725	0.5364	0.5486	0.5332
		DMk	0.7493	0.7526	0.6807	0.6066	0.5455
		AMI	0.4521	0.5053	0.4966	0.5334	0.5363
		CV	N/A	0.4797	0.4795	0.5327	0.5613
		TSM	0.5744	0.5839	0.5910	0.5466	0.5339
		CPF	0.7677	0.7682	0.7791	0.7720	0.7178
HOG50	purity	kTuple	0.5531	0.5534	0.5621	0.5149	0.4713
		DMk	0.6252	0.6117	0.6072	0.5654	0.4614
		AMI	0.3953	0.3892	0.4295	0.4448	0.4370
		CV	N/A	0.4983	0.5377	0.5672	0.5786
		TSM	0.5329	0.5206	0.5425	0.5260	0.5347
		CPF	0.6453	0.6483	0.6369	0.6351	0.6396
HOG50	F-measure	kTuple	0.4539	0.4273	0.4643	0.4490	0.4458
		DMk	0.5170	0.5334	0.4742	0.4647	0.3999
		AMI	0.2991	0.2719	0.2901	0.3254	0.3247
		CV	N/A	0.3809	0.4454	0.4553	0.5083
		TSM	0.4229	0.4164	0.4132	0.4304	0.4226
		CPF	0.5351	0.5411	0.5544	0.5441	0.5576
HOG80	purity	kTuple	0.5925	0.5841	0.5447	0.5381	0.4982
		DMk	0.6501	0.6381	0.6542	0.6452	0.5451
		AMI	0.4727	0.4721	0.4830	0.4957	0.4932
		CV	N/A	0.5655	0.5615	0.5979	0.6163
		TSM	0.5714	0.5840	0.5979	0.6053	0.5748
		CPF	0.6728	0.6691	0.6508	0.6721	0.6768
HOG80	F-measure	kTuple	0.4102	0.3838	0.4171	0.4570	0.4411
		DMk	0.4759	0.4187	0.4892	0.4379	0.4106
		AMI	0.2749	0.2792	0.2776	0.3287	0.3249
		CV	N/A	0.3594	0.3555	0.3941	0.4363
		TSM	0.3897	0.4063	0.3819	0.4069	0.4089
		CPF	0.5024	0.4624	0.4585	0.4614	0.4751
HOG100	purity	kTuple	0.5001	0.5404	0.5622	0.5320	0.4725
		DMk	0.6033	0.6233	0.6330	0.5651	0.4358
		AMI	0.4203	0.4437	0.4381	0.4197	0.4473
		CV	N/A	0.5101	0.4952	0.5646	0.5710
		TSM	0.4923	0.5132	0.5450	0.5416	0.5547
		CPF	0.6421	0.6359	0.6159	0.6295	0.6331
HOG100	F-measure	kTuple	0.3391	0.3785	0.4051	0.4436	0.4196
		DMk	0.4498	0.4582	0.4870	0.4163	0.3280
		AMI	0.2900	0.2985	0.2911	0.2801	0.3172
		CV	N/A	0.3438	0.3447	0.3741	0.4310
		TSM	0.3264	0.3329	0.3793	0.3827	0.4310
		CPF	0.4780	0.4491	0.4442	0.4509	0.4617

Generally, the CPF performance is very stable and better than other models in most cases, the sliding window size has a few effect on the CPF model. Namely, it achieves the best or near the best clustering result when the sliding window size is 2. That imples a great virtue of the CPF model. Because the longer sliding window size will consume much more computing resources including both time and space. In contrary, the DMk is unstable though it may be slightly better than the CPF occasionally.

As a result, a larger sliding window may not harvest a better clustering result. A shorter window size produces a shorter feature vector, which is greatly helpful in the large scale DNA processing because a shorter vector can reduce the computation overhead and speed up running time exponentially.

It is apparent that the word-frequency-only method (e.g. k-tuple, TSM, AMI and CV) performs worse than the other two hybrid methods (DMk and CPF) because they miss some useful information. In contrast, CPF considers more useful information and achieves better results.

### The running time

The Table 6 lists the average running time of six models at two stages against different *k* value on the dataset HOG80. Each of them is repeated 200 times to sum the running time. The running environment is as follows.

**Table 6:** **The runing time in seconds of the alignment-free models on the dataset HOG80**

Stage	Model	Size of sliding window				
		2	3	4	5	6
Building feature vector	kTuple	5.4848	5.9249	7.3474	13.1670	35.6224
	DMk	20.9364	22.3314	26.4229	42.6146	135.9681
	AMI	92.2859	121.9755	151.5307	181.6293	211.4968
	CV	N/A	17.8201	22.3311	33.8704	66.6548
	TSM	23.5510	23.7551	24.7025	24.2759	25.0376
	CPF	68.8990	69.3461	70.3552	72.0977	74.9368
k-means clustering	kTuple	20.7364	24.8182	57.3658	274.7188	1289.9252
	DMk	24.7368	58.8896	114.3778	513.7478	1721.9877
	AMI	17.0510	22.5570	20.3212	23.0417	19.7904
	CV	N/A	92.5585	147.1847	517.1461	1841.4720
	TSM	25.1845	23.8039	34.6972	48.2633	54.6024
	CPF	18.2820	34.2255	29.7729	41.6606	76.1415

CPU: Intel Core i7 (3.40GHz), RAM: 4.00GB, OS: Windows 7 (64bit professional edition).

At the feature vector building stage, the CPF model is slower than others except AMI. But at the k-means clustering stage, the CPF model is faster than others except AMI. Especially when *k* value is greater than 3, k-tuple, DMk and CV are tens times slower than CPF, TSM and AMI though the former three models are faster at the feature vector building stage.

It is a disadvantage for the CPF model to spend a long time on the feature vector building stage. But the CPF model runns very fast at the k-means clustering stage, If the feature vectors are not stored, namely, they are rebuilt in each clustering process, the total running time of CPF is near to AMI, but bigger than k-tuple, DMk and TSM. However, the feature vector of a DNA sequence is its inherent property, which is invariant. It can be built once and stored for repeated use. The CPF model is helpful to save time in the repeated clustering application.

## Discussion

There are a variety of features extracted from a raw DNA sequence, such as word frequency, classifications of nucleotide bases, position and so on. Different alignment-free models employ different features to build feature space. The dimension of a feature vector depends on the specific model. Generally, it varies from (*k*+1) to 4^*k*^. While *k*=6, it may be 4^6^=4096.

But, how much is adequate for DNA sequence comparison?

As far as I know, the theoretical boundary is not presented by now. But it is a fact that the longer feature vector may not guarantee the better clustering result. On the small size datasets, our experiments verified that the 12-dimension CPF model outperforms the 4096-dimension k-tuple model. Moreover, the experiments illustrate that the CPF model gets the best result when the sliding window is two, i.e. the dimension of a feature vector is 12 (3∗2^2^=12). Namely, the longer CPF vector (i.e. the longer sliding window) is not always better than the 12-dimension CPF vector. We have tested the CPF model on several datasets, the results show that CPF’s performance is steady. As a result, for not too long DNA sequences, the best configuration of CPF model is fixed.

However, the length of a DNA sequence is not very huge in our experiments, which is no more than 5000 characters. It believed that the 12-dimension vector is not adequate for huge DNA sequences. Obviously, if 10,000 characters size of sequences are compressed into 12-dimension vectors, too much information are lost so that they can not be effectively distinguished.

When the dataset grows large, all six models becomes worse. The best F-measure value declines from near 1.0 to near 0.5 when the family number of dataset grows from six to 100. All models’ F-measure value will less than 0.5 on the more larger dataset. Since the larger the dataset is, the more families may be overlapped eath other. That is a hard obstacle for alignment-free clustering models. It is a big challenge to solve the issue on the wild large scale datasets.

Consequently, it is still an problem for future research to estimate the optimal size of feature vectors for different size of DNA sequences. Our work illustrates that the 12-dimension CPF model can get an excellent clustering result for DNA sequences no more than 5000 characters.

## Conclusions

It is believed that an alignment-free model containing more useful information can achieve better data mining results. This paper presents the CPF model that employs the word frequency, position and nucleotide bases classification information from DNA sequences. The experimental results show that CPF is superior to other models, including k-tuple, DMk, TSM, AMI and CV. The following conclusions can be drawn from the experiments.

1. The hybrid information model is better than the model only based on word frequency.
2. For DNA sequences no more than 5000 characters, the preferred size of sliding windows for CPF is two, which provides a great advantage to promote system performance.
3. The CPF model is able to make an efficient stable performance and broad generalization.

In the future, we will perform the CPF model on a large scale DNA data to deeply observe DNA sequence similarity and mining relationships among them. And a more efficient clustering method will be presented to promote the clustering results on unknown DNA sequences.
